# Exploring the relationship between health literacy and fast food consumption: a population-based study from southern Iran

**DOI:** 10.1186/s12889-021-10763-3

**Published:** 2021-04-20

**Authors:** Azam Namdar, Mohammad Mehdi Naghizadeh, Marziyeh Zamani, Ali Montazeri

**Affiliations:** 1 Department of Community Medicine, School of Medicine, Fasa University of Medical Sciences, Fasa, Iran; 2 Non-Communicable Diseases Research Center, Fasa University of Medical Sciences, Fasa, Iran; 3 Department of Nutrition, School of Medicine, Jahrom University of Medical Science, Jahrom, Iran; 4 Population Health Research Group, Health Metrics Research Center, Institute for Health Sciences Research, ACECR, Tehran, Iran; 5 Faculty of Humanity Sciences, University of Science and Culture, Tehran, Iran

**Keywords:** Adults, Fast food, Health literacy, Iran, Reading skills, Decision-making

## Abstract

**Background:**

Health literacy (HL) may affect the consumption of fast food. We aimed to evaluate the effect of HL on fast food consumption among adult populations in Iran.

**Methods:**

We evaluated HL and fast food consumption in 421 adult participants with age range of 18–65 years old in Fasa, Fars Province, southern Iran. Two-step cluster and systematic sampling was performed to recruit the study sample. Data were collected using a fast food consumption checklist, and the Health Literacy Instrument for Adults (HELIA) by face-to-face interviews. Population data across groups with and without fast food intake were compared.

**Results:**

Most participants used fast food every few months (49.9%). People with low or unstable income consumed more fast food than others (*P* < 0.05). Sandwich and hotdog were the most consumed fast food (60.8%) followed by pizza (34.9%). Sausage and soda were the most seasoning food (66.7%). Most participants used fast food as dinner (67.9%) and with family (72.2%), suggesting the institutionalized consumption of this type of food in the family. Fun was the most frequent reason for the use of fast food (66.5%). Most participants completely knew about the raw materials for fast food and their adverse effects. Finally, we found that overall health literacy was lower among those who used fast food than those who did not. Consumed fast food (68.16 ± 23.85 vs. 73.15 ± 20.15; *p* = 0.021). This difference was also observed for some components of health literacy including reading skills, and decision-making subscales.

**Conclusions:**

The findings suggest there is a negative relationship between general health literacy and fast food consumption indicating that who possess lower level of health literacy is likely to consume more fast food. Specifically, the findings suggest that reading skills, and decision-making (behavioral intention) are more associated with decreased or increased fast food intake.

**Supplementary Information:**

The online version contains supplementary material available at 10.1186/s12889-021-10763-3.

## Background

With the advance of industry and technology, during the past four decades, food consumption patterns and nutritional habits in the Middle East have changed significantly. This change directed nutrition transition from traditional foods to western foods, which are characterized by high fat, high cholesterol, high sodium and low fiber diet. In addition, food consumption in restaurants and fast-food has become increasingly common [1, 2]. Fast food is defined as a convenience food purchased in self-service or carry-out eating venues without wait service [3]. This type of food can induce several health problems such as body weight gain. In this regard, most people do not know about the harmful effects of this type of food [4]. Despite severe adverse health effects, fast food consumption has increased gradually due to the increase in the number of women working, changes in the family structure, worldwide urbanization, long working hours, and rapid growth of fast food industries and restaurants [5]. Thus, it seems that fast food consumption is becoming a major public health problem worldwide. As such many governments are seeking to find out ways that could reduce fast food consumption. In this regard some investigators proposed that if we could increase health literacy among populations, then it might be possible to reduce fast food consumption much easier [6].

Health literacy is considered as one of the most important skills to control people’s health [7]. Health literacy is the ability to acquire, process, and conceive basic health-related information and services [8]. Based on the definition provided by the World Health Organization (WHO), HL is a complex of social and cognitive abilities to acquire, understand, and apply health-related information to promote health and maintain good health [9]. It has been confirmed that people with higher levels of HL have more information about their health status [10]. However, one specific form of health literacy is nutrition literacy. Nutrition literacy reflects the ability to access, interpret, and use nutrition information and exactly focuses on HL skills related to food consumption [11].

Previous research showed that high food literacy was associated with increased consumption of fruits and vegetables; and low level of food and nutrition literacy was associated with nutritional inadequacy in the school-age children [12–14]. In addition, it has been suggested that significantly higher nutrition knowledge exists among the persons with adequate HL [15]. Moreover, studies have shown that a significantly inverse relationship exists between HL and self-care including physical activity and diet, between limited HL and high BMI, and between low HL and overweight and obesity in children and adolescents [16–18]. Higher level of health literacy leads to better nutrition status during pregnancy and increasing the level of health literacy can raise the nutritional behaviors [19, 20].

However, it is argued that most investigations on health literacy did not explicitly focus on food or nutrition and thus, dietetics practitioners often remain unaware of their clients’ HL level [6, 21]. Overall it is believed that the association between health literacy and fast food consumption is unclear and limited evidence exist on the topic [22]. Thus, the present study was conducted to evaluate the relationship between HL and fast food consumption among a sample of adult population.

## Methods

### Design

The present cross-sectional study was conducted on a sample of adult population (aged 18–65 years old) in Fasa, Fars Province, southern Iran in 2018. The study was approved by the Ethics Committee of Fasa University of Medical Sciences (IR.FUMS.REC.2017.255).

### Sample size and sampling

The sample size was determined using the Cochran’s formula for infinite populations (n = z^2^pq/d^2^, p = q = 0.5, z = 1.96, d = 0.05) [23]. Based on this formula, the required size was estimated at 384, which was then increased to 423 with the probability of 10% attrition. The sampling method was stratified cluster design that conducted in two steps. Clinics and health centers were considered as the clusters. In the first stage, from 11 regions 3 clinics and 8 health centers were selected. In the next stage, systematic sampling was performed and, a list of households (as a unit of the sampling) and the number of samples in each stratum were estimated. The inclusion criteria were: adults aged 18 to 65 years old (male or female), being Iranian, residence in Fasa during the research period, and being literate (ability to read and write). The exclusion criteria were: adults having diet restrictions, and refusal to give informed consent.

### Instruments

1. Health literacy: It was measured using the Health Literacy Instruments for Adults-HELIA [24]. The HELIA measures five dimensions: “access” (items 1–6), “reading” (items 7–10), “understanding” (items 11–17), “appraisal” (18–21), and “decision-making/behavioral intention” (items 22–33). Scores on HELIA are classified into four categories: inadequate and problematic (which together define “limited health literacy”), sufficient and excellent (which together define “desired health literacy”). Scores on the HELIA range from 0 to 100 that represent the following criteria: 0–50: inadequate, 50.1–66: problematic, 66.1–84: sufficient, and 84.1–100 excellent. The psychometric properties of the HELIA are well documented [24, 25]. The questionnaire is provided as Additional file 1.

2. Fast food consumption: A checklist was used to collect data on fast food intake by the respondents. Based on the study objectives it was specifically developed by the research team and contained 20 items including type of fast food consumed and how often they consume fast foods. The checklist was completed for each respondent by trained interviewers. By fast food, we meant different sandwiches; hamburgers, cheeseburgers, and other burgers; fried fish and shrimp; hot dog; meat or chicken steak; French fries; fried chicken; tacos (a Mexican dish); pizzas; and snacks, which are usually prepared in restaurants and outside the home. If fast food was consumed at least once a month, the person was considered to be a user; otherwise, he/she was considered as a non-user. Finally, consuming fast food less than once a week was called “low use”, 1–2 times a week was called “moderate use”, and more than twice a week was labeled as “excessive use” [26]. The last part of the checklist included items on demographic information such as age, sex, education, occupation, marital status and self-reported weight, height and income. Income was categorized as low, intermediate, and high. A panel of experts qualitatively assessed the content and face validity of the checklist [Additional file 2].

3. Body Mass Index (BMI) was calculated by self-reported weight and height using the following formula: weight in kg/height in m^2^ [27]. Based on WHO classification, BMI ≥25 kg/m^2^ was considered as overweight and obese [28].

### Data collection

Data were collected by a team of trained interviewers. As such they were given necessary training on how to communicate and how to record the information. All interviews were conducted at participants’ homes. One of us (AN) was responsible for monitoring the data collection processes to ensure the accuracy of data and information collected.

### Data analysis

Normality of all data were checked by Kolmogorov-Smirnov test. Data were expressed as mean, standard deviation (SD), frequency, and percentage. The Chi-squared test was used for group comparison (between with and without of fast food intake). The score for the HELIA was compared across the groups using the t-test. Moreover, odds ratio and the confidence intervals were calculated using logistic regression analysis. The significance level was set at < 0.05 in all instances. The data were analyzed using IBM SPSS 24 (IBM SPSS CO., Armonk, NY).

## Results

In all 421 participants were entered into the study. Of these 210 individuals were fast food users and the remaining 211 were non-users. The mean age of participants was 37.3 ± 11.5 years. The mean age of fast food users was 36.2 ± 11.9 years and that of non-users was 38.3 ± 11.1 years (*p* = 0.056). The mean BMI was found to be 25.1 ± 5.1 kg/m^2^. The demographic characteristics of the study sample are presented in Table 1.

**Table 1:** Demographic variables and fast food consumption in the study sample

	All (***n*** = 421)	Users (***n*** = 210)	Non-users (***n*** = 211)	***P***-value*
	No. (%)	No. (%)	No. (%)	
**Gender**				0.365
Female	294 (69.8)	151 (71.9)	143 (67.8)	
Male	127 (30.2)	59 (28.1)	68 (32.2)	
**Marital status**				0.554
Single	105 (24.9)	55 (26.2)	50 (23.7)	
Married	316 (75.1)	155 (73.8)	161 (76.3)	
**Education**				0.918
< Secondary education	77 (18.3)	38 (18.1)	39 (18.5)	
≥ Secondary education	344 (81.7)	172 (81.9)	172 (81.5)	
**Income**				0.023
low	308 (59.8)	145 (69.4)	163 (79.1)	
Intermediate/high	107 (20.8)	64 (30.6)	43 (20.9)	
**BMI (kg/m**^**2**^)*				
< 25	223 (53.0)	116 (55.2)	107 (50.7)	0.325
≥ 25	198 (47.0)	94 (44.8)	104 (49.3)	
**Age**				
Mean (SD)^c^	37.3 (11.5)	38.3 (11.1)	36.2 (11.9)	0.056

*BMI* Body Mass Index (kilograms/ Square meters), *SD* Standard deviation

* Derived from Chi-square test and independent-samples t-test as appropriate

Based on the findings, 49 persons (11.6%) had a fast food membership card. Different types of sandwiches and hot dog had the highest rate of intake (60.8%, *n* = 256) and steak ranked the least (3.8%, *n* = 16). Moreover, 349 people (82.9%) consumed sauces with fast food and 281 (66.7%) drank soft beverages with it. The motivation for fast food consumption was enjoyment and fun for 280 people (66.5%). Furthermore, 116 (27.6%) consumed it, because they were busy and had little time to prepare food at home. Based on the definitions, 16 people (8.3%) were excessive users, 295 (70.8%) were low users, and the rest were moderate users (*n* = 110, 20.9%). The detailed findings on pattern of fast food consumption are presented in Table 2.

**Table 2:** Pattern of fast food consumption in the study sample

Variable		No	%
Fast food consumption frequency	More than twice a week	16	3.8
	1–2 times a week	107	25.4
	Once or twice a month	88	20.9
	Less than once a month	210	49.9
Membership card	Yes	49	11.6
	No	372	88.4
Types of sandwich and hot dog	Yes	256	60.8
	No	165	39.2
Pizza	Yes	147	34.9
	No	274	65.1
Other (snacks, fried foods …)	Yes	80	19.0
	No	341	81.0
Popularity	Any types of sandwiches	178	42.3
	Hot dog	4	1.0
	Pizza	114	27.1
	French fries	38	9.0
	Steak	16	3.8
	Fried chicken	31	7.4
	Fried fish and shrimp	40	9.5
Sauces consumption	Yes	349	82.9
	No	72	17.1
Soft drinks	Yes	281	66.7
	No	140	33.3
Meal	Breakfast	1	0.2
	Lunch	113	26.8
	Dinner	286	67.9
	Supper	21	5.0
Companions	Family	304	72.2
	Friends	96	22.8
	Alone	21	5.0
Motivation: Enjoyment and fun	Yes	280	66.5
	No	141	33.5
Motivation: Ease of access	Yes	36	8.6
	No	385	91.4
Motivation: Busy and time constrain to prepare food at home	Yes	116	27.6
	No	305	72.4
Place of use	Outside (restaurant)	233	55.3
	Home	188	44.7
Knew the fast food ingredients	Yes	292	69.4
	No	129	30.6
Priority factors in choosing fast food	Hygiene	321	76.2
	Diversity	90	21.4
	Price	10	2.4
Aware of the harmfulness of fast food	Yes	381	90.5
	No	40	9.5

The findings for the HELIA are presented in Table 3. The mean health literacy score was 70.65 (SD = 22.20). In all 161 participants (48.7%) had limited HL (inadequate and problematic).

**Table 3:** Health literacy score by items and levels

	**Health literacy by items**	**Never**	**Rarely**	**Sometimes**	**Usually**	**Always**
		**No. (%)**	**No. (%)**	**No. (%)**	**No. (%)**	**No. (%)**
**Reading**	Reading educational materials about health (booklets, pamphlets, and leaflets) is easy for me.	13 (3.1)	14 (3.3)	96 (22.8)	159 (37.8)	139 (33.0)
	Reading written instructions from doctors, dentists and health workers about my illness is easy for me.	15 (3.6)	39 (9.3)	88 (20.9)	156 (37.1)	123 (29.2)
	Reading medical and dental forms (such as admissions, consent, filing, etc. in hospitals and medical centers) is easy for me.	14 (3.3)	38 (9.0)	98 (23.3)	143 (34.0)	128 (30.4)
	Reading leaflets and instructions for laboratory testing, ultrasound or radiology is easy for me.	12 (2.9)	52 (12.4)	83 (19.7)	144 (34.2)	130 (30.9)
**Access to information**	I can find health information from different sources when I need such information.	25 (5.9)	44 (10.5)	132 (31.4)	142 (33.7)	78 (18.5)
	I can find health information about healthy eating.	10 (2.4)	39 (9.3)	105 (24.9)	158 (37.5)	109 (25.9)
	I can find health information on mental health such as depression and stress.	29 (6.9)	74 (17.6)	117 (27.8)	127 (30.2)	74 (17.6)
	I can find health information about a specific disease when I need to.	17 (4.0)	46 (10.9)	127 (30.2)	133 (31.6)	98 (23.3)
	I can find health information for some health problems and diseases such as high blood pressure, high blood sugar and high lipid levels.	17 (4.0)	56 (13.3)	86 (20.4)	154 (36.6)	108 (25.7)
	I can find health information about harmful effects of tobacco and smoking.	15 (3.6)	31 (7.4)	65 (15.4)	132 (31.4)	178 (42.3)
**Understanding**	I can understand the recommendations for a healthy diet.	5 (1.2)	14 (3.3)	48 (11.4)	143 (34.0)	211 (50.1)
	I can understand when my physician explains about my illness.	2 (0.5)	11 (2.6)	41 (9.7)	135 (32.1)	232 (55.1)
	I can understand the meaning when reading medical forms (such as admissions, consents, filings, etc.) in hospitals and health centers.	9 (2.1)	34 (8.1)	74 (17.6)	134 (31.8)	170 (40.4)
	I can understand signage guidelines in hospitals, clinics and health centers.	6 (1.4)	13 (3.1)	66 (15.7)	129 (30.6)	207 (49.2)
	I can understand drug information on labels.	7 (1.7)	22 (5.2)	33 (7.8)	109 (25.9)	250 (59.4)
	I can understand the risks, and benefits of drugs prescribed by my physician.	12 (2.9)	27 (6.4)	58 (13.8)	142 (33.7)	182 (43.2)
	I can understand written information before testing, ultrasound or radiology.	33 (7.8)	44 (10.5)	80 (19.0)	134 (31.8)	130 (30.9)
**Appraisal**	I can evaluate health-related information on the Internet.	56 (13.3)	79 (18.8)	119 (28.3)	102 (24.2)	65 (15.4)
	I can evaluate health-related information broadcast on television and radio.	11 (2.6)	45 (10.7)	108 (25.7)	161 (38.2)	96 (22.8)
	I can assess the accuracy of health-related recommendations I receive from relatives and friends.	15 (3.6)	54 (12.8)	110 (26.1)	138 (32.8)	104 (24.7)
	I can communicate trusted health information to others	14 (3.3)	26 (6.2)	92 (21.9)	128 (30.4)	161 (38.2)
**Decision-making/ behavioral intention**	When facing an illness, I know where to go or with who me to speak.	6 (1.4)	40 (9.5)	85 (20.2)	133 (31.6)	157 (37.3)
	When physician suggests that I should take antibiotic capsules three times a day I know that I should take one tablet every 8 h.	6 (1.4)	19 (4.5)	46 (10.9)	124 (29.5)	226 (53.7)
	I do not cut my medications without my physician’s permission, even if symptoms disappear.	13 (3.1)	33 (7.8)	84 (20.0)	121 (28.7)	170 (40.4)
	If anyone from my first-degree relatives develops cancer (such as prostate, breast, cervix, colon, etc.), I see a doctor to examine me.	28 (6.7)	80 (19.0)	72 (17.1)	102 (24.2)	139 (33.0)
	I avoid doing or eating things that increase my blood pressure.	13 (3.1)	43 (10.2)	98 (23.3)	265 (62.9)	2 (0.5)
	I visit my physician for regular checkups.	61 (14.5)	101 (24.0)	94 (22.3)	74 (17.6)	91 (21.6)
	I am health-conscious in any situation.	6 (1.4)	33 (7.8)	100 (23.8)	160 (38.0)	122 (29.0)
	If needed, I ask my physician or health care team questions about my disease.	11 (2.6)	59 (14.0)	78 (18.5)	145 (34.4)	128 (30.4)
	I buy dairy products (milk, yoghurt, cheese, etc.) according to their fat percentage.	13 (3.1)	59 (14.0)	76 (18.1)	129 (30.6)	144 (34.2)
	I avoid using substances that increase my weight.	74 (17.6)	85 (20.2)	130 (30.9)	132 (31.4)	0 (0.0)
	I use a seat belt when driving.	6 (1.4)	23 (5.5)	52 (12.4)	105 (24.9)	235 (55.8)
	I consider the food labels when shopping	9 (2.1)	36 (8.6)	86 (20.4)	144 (34.2)	146 (34.7)
	**Health literacy by dimensions**	**Total**	**Inadequate**	**Problematic**	**Sufficient**	**Excellent**
		**Mean (SD)**	**No. (%)**	**No. (%)**	**No. (%)**	**No. (%)**
	**Reading**	66.08 (21.51)	115 (27.3)	72 (17.1)	154 (36.6)	80 (19.0)
	**Access to information**	78.65 (18.33)	38 (9.0)	55 (13.1)	135 (32.1)	193 (45.8)
	**Understanding**	64.62 (12.51)	124 (29.5)	85 (20.2)	134 (31.8)	78 (18.5)
	**Appraisal**	68.07 (17.04)	73 (17.3)	93 (22.1)	179 (42.5)	76 (18.1)
	**Decision making**	69.85 (15.27)	42 (10.0)	119 (28.3)	182 (43.2)	78 (18.5)
	**Total score**	70.65 (22.20)	92 (21.9)	69 (16.4)	136 (32.3)	124 (29.5)

Table 4 compares the mean HELIA scores of users and non-users. The results revealed that the mean HELIA score was significantly lower in users () compared to non-users (68.16 ± 23.85 vs. 73.15 ± 20.15; *p* = 0.021). The same was true for reading, and decision-making subscales (*p* < 0.001, and *p* = 0.018, respectively).

**Table 4:** Comparing health literacy between fast food users and no-users

	Users (***n*** = 110)	Non-users (***n*** = 211)	***P***-value*
	Mean (SD)	Mean (SD)	
**Reading**	69.72 (20.30)	62.46 (22.10)	< 0.001
**Access**	80.22 (16.44)	77.08 (19.95)	0.089
**Understanding**	64.43 (20.03)	64.81 (22.94)	0.855
**Appraisal**	69.78 (15.89)	66.36 (17.98)	0.111
**Decision making**	71.75 (14.04)	67.96 (16.21)	0.018
**Total score**	73.15 (20.15)	68.16 (23.85)	0.026

* Derived from t test

The results obtained from logistic regression analysis (after controlling for confounding variables) showed that by 1 score increase in health literacy, the odds of fast food intake was reduced by 1% (OR = 0.990, 95% CI = 0.981–0.999). This relationship was also observed for the ability of reading (OR = 0.985, 95% CI = 0.975–0.993) and decision-making (OR = 0.986, 95% CI = 0.072–0.999). The results for both unadjusted and adjusted odds ratio for fast food intake are shown in Table 5.

**Table 5:** The association between health literacy and fast food intake obtained from logistic regression analysis

	Univariate analysis		Adjusted analysis*	
	OR (95% CI)	***P***-value**	OR (95% CI)	***P***-value**
**Reading**	0.984 (0.975–0.993)	0.001	0.985 (0.975–0.995)	0.004
**Access**	0.991 (0.980–1.001)	0.080	0.992 (0.981–1.004)	0.191
**Understanding**	1.001 (0.992–1.010)	0.858	1.001 (0.992–1.011)	0.781
**Appraisal**	0.988 (0.977–0.999)	0.040	0.991 (0.980–1.004)	0.166
**Decision making**	0.984 (0.971–0.996)	0.011	0.986 (0.972–0.999)	0.049
**Total score**	0.990 (0.981–0.998)	0.022	0.990 (0.981–0.999)	0.045

*OR* Odds ratio

**Adjusted for age, gender, education, income and BMI

*Derived from logistic regression analysis

**Fig. 1:**
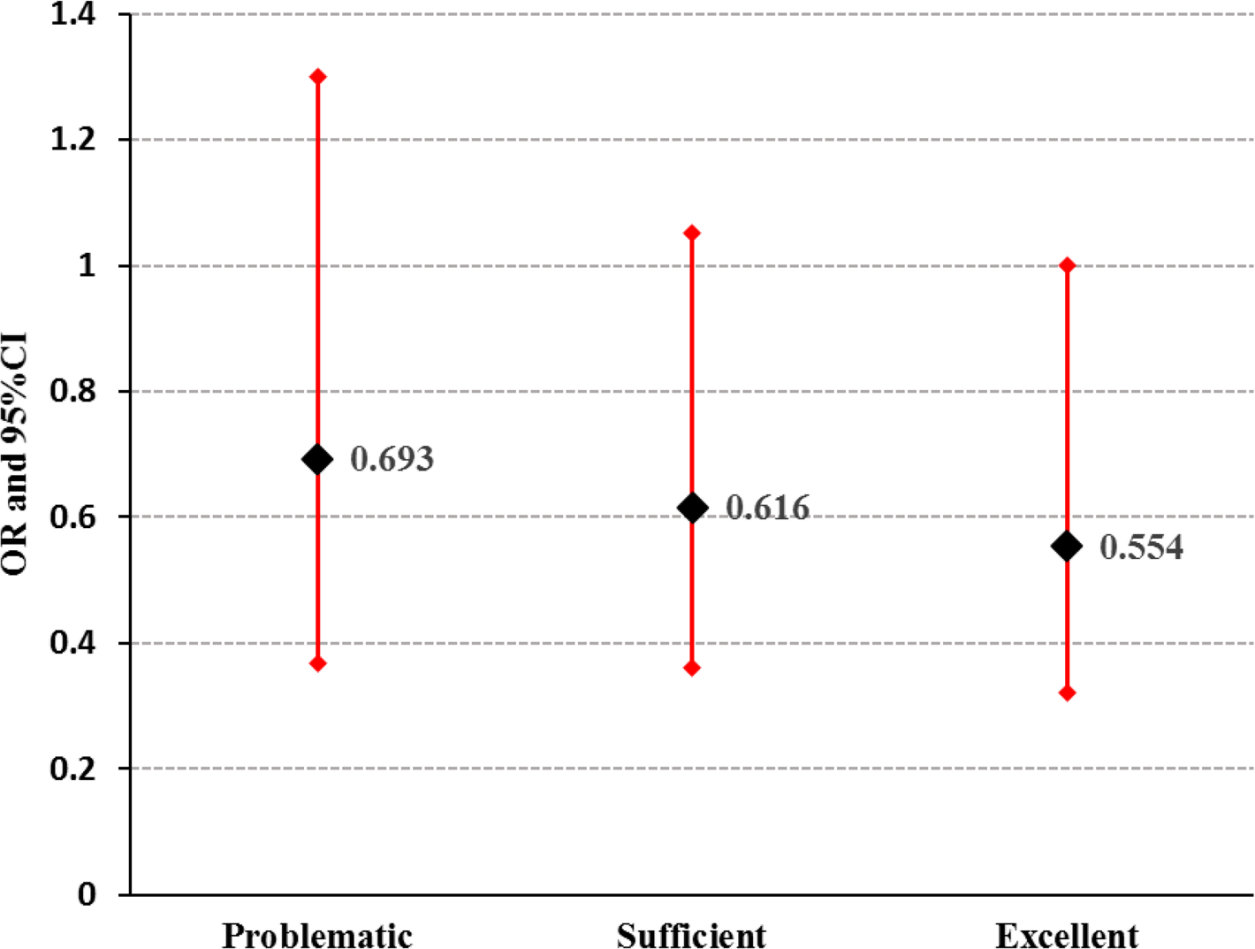
The results obtained from logistic regression analysis for odds ratio (OR) with 95% confidence intervals (CI) of consuming fast food in people with different levels of health literacy. The odds of using fast food was decreasing when health literacy increased. The analysis was adjusted for age, gender, education, income, and BMI. Inadequate health literacy was set as the reference group

Finally, when the odds of fast food intake in those with different level of health literacy (problematic, sufficient, and excellent) was compared to those with inadequate health literacy as the reference group, a decreasing dose-response was observed (OR for problematic = 0.693, *p* = 0.253; OR for sufficient: 0.616, *p* = 0.076; and OR for excellent = 0.554, *p* = 0.034). The findings are depicted in Fig. 1.

## Discussion

The present study examined the relationship between HL and fast food consumption among a sample of the adult population, and the findings showed that about 50% of the study participants were using fast food regularly. They used fast food as dinner and with their family, suggesting the institutionalized consumption of this type of food in the family. Also, 11.6% of the respondents indicated that they had a fast food membership card, indicating a tendency for the repeated intake of fast food in the future. In addition, people with lower income used more fast food than other peoples.

In general, the finding from the current study is alarming. There is evidence that in Iran the nutritional transition is accelerating towards an increased consumption of fast food, followed by an increase in the prevalence of chronic diseases [29]. It is argued that developing countries are experiencing major changes in their nutrition as a result of a significant increase in per capita income [30, 31]. As such the process of eating has shifted toward food away from home, and spending an increasing share of food expenditures on food away from home [32]. Similarly, worldwide consumption of food away from home has grown significantly in the past two decades [33, 34].

The findings also showed that HL in those who used fast food was less than those who did not. This difference specifically was observed for reading skills, appraisal ability and decision-making. The findings from this study confirm that HL has a close relationship with health-related behavior such as fast food consumption. In addition, the findings highlights the fact that nutrition literacy is a specific component of health literacy that reflects the ability to access, interpret, and use nutrition-related information [6, 35].

Given the importance of HL and its relationship with fast food consumption, it seems there are limited studies on the topic. However, several studies have demonstrated that there is an association between fast food consumption and obesity as a public health problem [36, 37]. In a recent study on the relationship between HL and nutritional practice in high school adolescents of Tehran, capital of Iran, 74.5% of the adolescents had inadequate and problematic health literacy, and 68% had unsatisfactory nutritional practice. In addition, similar to our findings, nutritional practice was improved by increasing HL [38].

We found that reading skills and decision-making were important components of health literacy and had associations with fast food consumption. Linnebur also showed that limited HL was associated with students’ inability to read and understand food labels [39]. Perhaps this is also true for the general population especially for people with lower education level.

### Strengths and limitations

The study had several strengths. The main strength was the focus on HL and its relationship with fast food consumption that received less attention. The sampling method was another strength of this study since it was a population-based study. In addition, we used a well-developed instrument for HL that covers public health related items. Finally, the completion of the questionnaires through structured interviews rather than self-reported was another strength. This study however had some limitations. One limitation of this study was the use of a general HL that is inadequate for studies on nutrition because it does not focus on food or nutrition literacy. One should note that although very related, measuring health literacy, food or nutrition literacy are needing different instruments [11, 40, 41]. Therefore, we recommend the further investigations use specific food or nutrition literacy instruments. We were unfortunate to have validated Persian instruments such as the Nutrition Literacy Assessment Instrument [42] in Iran at the study commence. Secondly, potential bias related to the sampling method (cluster sampling and systematic sampling) should be acknowledged. We used the household list as the only available framework for systematic sampling. This list was provided by health centers and was not updated since 5 years ago. Thus, lack of an up-to-date sampling framework could be a weakness. Thirdly since our study was cross-sectional, we could not establish a causal inference. Finally, the study participants were from the southern region of Iran only, and so our results could not represent other regions of the country, therefore, more studies, based on large national representative samples are needed to better understand the relationship between HL and fast food intake in order to implement appropriate interventions. Educational interventions are recommended to improve HL with emphasis on increasing NL.

## Conclusion

The findings suggest there is a negative relationship between health literacy and fast food consumption indicating that who possess lower level of health literacy is likely to consume more fast food. Specifically, the findings suggest that reading skills, and decision-making (behavioral intention) are more associated with decreased or increased fast food intake.

## Supplementary Information

**Additional file 1.** The HELIA English version.

**Additional file 2.** Namdar Fast Food Consumption Checklist.

## Data Availability

The data are available from the corresponding authors.

## Abbreviations

HELIA: Health Literacy Instrument for Adults
HL: Health literacy
NL: Nutrition literacy
BMI: Body Mass Index
SD: Standard Deviation

## References

[1] Farazmand H, Hallafi H (2016). The demand for away from home food in Iranian households: An application of box-cox double hurdle model. Econ Model.

[2] Musaiger AO (2004). Overweight and obesity in the eastern Mediterranean region: can we control it?. East Mediterr Health J.

[3] Mohammadbeigi A, Asgarian A, Moshir E, Heidari H, Afrashteh S, Khazaei S, Ansari H (2018). Fast food consumption and overweight/obesity prevalence in students and its association with general and abdominal obesity. J Prev Med Hyg.

[4] Burgoine T, Sarkar C, Webster CJ, Monsivais P (2018). Examining the interaction of fast-food outlet exposure and income on diet and obesity: evidence from 51,361 UK biobank participants. Int J Behav Nutr Phys Act.

[5] An R (2016). Fast-food and full-service restaurant consumption and daily energy and nutrient intakes in US adults. Eur J Clin Nutr.

[6] Carbone ET, Zoellner JM (2012). Nutrition and health literacy: a systematic review to inform nutrition research and practice. J Acad Nutr Diet.

[7] World Health Organization (1997). The Jakarta Declaration On Leading Health Promotion into the 21st century.

[8] Truman E, Bischoff M, Elliott C (2019). Which literacy for health promotion: health, food, nutrition or media?. Health Promot Int.

[9] Yusefi A, Ebrahim Z, Bastani P, Najibi M, Radinmanesh M, Mehrtak M (2019). Health literacy status and its relationship with quality of life among nurses in teaching hospitals of Shiraz University of Medical Sciences. Iran J Nurs Midwifery Res.

[10] Walker J, Pepa C, Gerard PS (2010). Assessing the health literacy levels of patients using selected hospital services. Clin Nurse Spec.

[11] Velardo S (2015). The nuances of health literacy, nutrition literacy, and food literacy. J Nutr Educ Behav.

[12] Burrows TL, Lucas H, Morgan PJ, Bray J, Collins CE (2015). Impact evaluation of an after-school cooking skills program in a disadvantaged community: Back to basics. Can J Diet Pract Res.

[13] Utter J, Denny S, Lucassen M, Dyson B (2016). Adolescent cooking abilities and behaviors: associations with nutrition and emotional well-being. J Nutr Educ Behav.

[14] Doustmohammadian A, Omidvar N, Keshavarz-Mohammadi N, Eini-Zinab H, Amini M, Abdollahi M, Amirhamidi Z, Haidari H (2020). Low food and nutrition literacy (FNLIT): a barrier to dietary diversity and nutrient adequacy in school age children. BMC Research Notes.

[15] Gibbs HD, Ellerbeck EF, Befort C, Gajewski B, Kennett AR, Yu Q, Christifano D, Sullivan DK (2016). Measuring nutrition literacy in breast cancer patients: development of a novel instrument. J Cancer Educ.

[16] Ghaedi M, Banihashemi F, Latifi M, Soleymaninejad M (2016). The relationship between health literacy and self-care among patients with type 2 diabetes residing in the city of Bastak. Iran J Endocrinol Metab.

[17] Al-Ruthia YS, Balkhi B, AlGhadeer S, Mansy W, AlSanawi H, AlGasem R, AlMutairi L, Sales I (2017). Relationship between health literacy and body mass index among Arab women with polycystic ovary syndrome. Saudi Pharmaceutical J.

[18] Lam LT, Yang L (2014). Is low health literacy associated with overweight and obesity in adolescents: an epidemiology study in a 12–16 years old population, Nanning, China, 2012. Arch Public Health.

[19] Sajadi H, Hoseinpour N, Sharifian Sani M, Mahmoodi Z (2016). Health literacy and life style status in married rural women of Izeh, Iran. J Health.

[20] Ahmadzadeh Sani T, Vahedian-Shahroodi M, Tehrani H, Esmaily H (2019). Relationship between health literacy and nutrition among middle-aged women. J Health Literacy.

[21] Makiabadi E, Kaveh MH, Mahmoodi MR, Asadollahi A, Salehi M (2019). Enhancing nutrition-related literacy, knowledge and behavior among university students: a randomized controlled trial. Int J Nutr Sci.

[22] Ludmilla FW-S, Rikard RV (2017). Review of health literacy and nutrition-related research in the Middle East. Arab J Nutr Exer (AJNE).

[23] Cochran WG (1977). Sampling Techniques. 3, editor.

[24] Montazeri A, Tavousi M, Rakhshani F, Azin SA, Jahangiri K, Ebadi M, Naderimagham S, Solimanian A, Sarbandi F, Motamedi A (2014). Health literacy for Iranian adults (HELIA): development and psychometric properties. Payesh (Health Monitor).

[25] Tavousi M, Haeri-Mehrizi A, Rakhshani F, Rafiefar S, Soleymanian A, Sarbandi F, Ardestani M, Ghanbari S, Montazeri A (2020). Development and validation of a short and easy-to-use instrument for measuring health literacy: the health literacy instrument for adults (HELIA). BMC Public Health.

[26] Pereira MA, Kartashov AI, Ebbeling CB, Van Horn L, Slattery ML, Jacobs DR, Ludwig DS (2005). Fast-food habits, weight gain, and insulin resistance (the CARDIA study): 15-year prospective analysis. Lancet.

[27] World Health Organization (2000). Obesity: preventing and managing the global epidemic. Report of a WHO Consultation (WHO Technical Report Series 894).

[28] Ulijaszek SJ (2003). Obesity: preventing and managing the global epidemic. Report of a WHO consultation. WHO technical report series 894. Pp. 252. (World Health Organization, Geneva, 2000.) SFr 56.00, ISBN 92-4-120894-5, paperback. J Biosoc Sci.

[29] Ghassemi H, Harrison G, Mohammad K (2002). An accelerated nutrition transition in Iran. Public Health Nutr.

[30] Chang H-H, Yen ST (2010). Off-farm employment and food expenditures at home and away from home. Eur Rev Agric Econ.

[31] Akbay C, Tiryaki GY, Gul A (2007). Consumer characteristics influencing fast food consumption in Turkey. Food Control.

[32] Bai J, Wahl TI, Lohmar BT, Huang J (2010). Food away from home in Beijing: effects of wealth, time and “free” meals. China Econ Rev.

[33] Mancino L, Todd J, Lin B-H (2009). Separating what we eat from where: measuring the effect of food away from home on diet quality. Food Policy.

[34] Liu M (2011). Food expenditures away from home by type of meal and by facility. Master Thesis, University of Tennessee.

[35] Watson WL, Chapman K, King L, Kelly B, Hughes C, Yu Louie JC, Crawford J, Gill TP (2013). How well do Australian shoppers understand energy terms on food labels?. Public Health Nutr.

[36] Fung C, McIsaac J-LD, Kuhle S, Kirk SF, Veugelers PJ (2013). The impact of a population-level school food and nutrition policy on dietary intake and body weights of Canadian children. Prev Med.

[37] Dunn RA, Sharkey JR, Horel S (2012). The effect of fast-food availability on fast-food consumption and obesity among rural residents: an analysis by race/ethnicity. Econ Human Biol.

[38] Saeedy Golluche F, Jalili Z, Tavakoli R, Ghanbari S (2017). The study of relationship between health literacy and nutritional practice in high school adolescents in Tehran. Iran J Health Educ Health Promot.

[39] Linnebur LA, Linnebur SA (2018). Self-administered assessment of health literacy in adolescents using the newest vital sign. Health Promot Pract.

[40] Truman E, Lane D, Elliott C (2017). Defining food literacy: a scoping review. Appetite.

[41] Vettori V, Lorini C, Milani C, Bonaccorsi G (2019). Towards the implementation of a conceptual framework of food and nutrition literacy: providing healthy eating for the population. Int J Environ Res Public Health.

[42] Gibbs HD, Ellerbeck EF, Gajewski B, Zhang C, Sullivan DK (2018). The Nutrition Literacy Assessment Instrument is a valid and reliable measure of nutrition literacy in adults with chronic disease. J Nutr Educ Behav.

