# Supplementary material for: Exploring the relationship between health literacy and fast food consumption: a population-based study from southern Iran
Source: BMC Public Health. 2021 Apr 20;21:757. doi: 10.1186/s12889-021-10763-3 (PMC8056591; doi:10.1186/s12889-021-10763-3)
Supplement: Supplementary file 2 — Additional file 2. Namdar Fast Food Consumption Checklist. [file 12889_2021_10763_MOESM2_ESM.doc]

**Fast Food Consumption Checklist**

Dear respondent, please answer the following questions carefully and honestly. Thank you in advance for your cooperation.

Note: by Fast food we mean sandwiches, burgers, cheeseburgers, and other types of burgers, fried fish and shrimp, hot dogs, beef and chicken steak, French fries, fried chicken, tacos (Mexican food), pizza and snacks, that are usually prepared outside the home and in restaurants.

1. Have you ever eaten fast food?

Yes ❑ Never ❑

1. Do you have a subscription card for fast food restaurants?

Yes ❑ No ❑

1. Which types of fast food do you often eat?

Sandwiches ❑ Hot dogs ❑ Pizza ❑ French fries ❑ Beef and chicken steak ❑ Fried chicken ❑ Fried fish and shrimp ❑

1. What is your favorite fast food item?

Sandwiches ❑ Hot dogs ❑ Pizza ❑ French fries ❑ Beef and chicken steak ❑ Fried chicken ❑ Fried fish and shrimp ❑

1. Do you use any condiments such as sauces along with fast food?

Yes ❑ No ❑

1. Do you use soft drinks along with fast food?

Yes ❑ No ❑

1. How often do you eat fast food?

Daily ❑ Every other day ❑ Once a week ❑ Once or twice a week ❑ Three times a week or more ❑ Once every two weeks ❑ Once a month ❑ Once every few months ❑

1. Which meal do you usually eat fast food?

Breakfast ❑ Lunch ❑ Dinner ❑ Afternoon Snack ❑

1. Who do you usually eat fast food with?

Family ❑ Friends ❑ Alone ❑

1. Why do you eat fast food?

Being delicious ❑ Low cost ❑ Easy access ❑ Family members are busy with work or study ❑ Lack of sufficient time for cooking ❑ Time and space constraints for food preparation ❑ Having fun with friends ❑

1. Are you aware of the ingredients used in the fast food preparation?

Yes ❑ No ❑

1. Are you aware of the disadvantages of fast food?

Yes ❑ No ❑

1. Gender

Female ❑ Male ❑

1. Marital status

Single ❑ Married ❑

1. Educational level

Primary ❑ Secondary ❑ Higher ❑

1. Occupation

Housewife ❑ Student ❑ Employed ❑ Unemployed ❑ Retired ❑

1. Income

Low ❑ Intermediate ❑ High ❑

1. Age…………years
2. Weight (kilograms)…………….
3. Height (meters)…………………

**Thank you for responding**

© Namdar et al. 2018
